# Knowlesi malaria in Vietnam

**DOI:** 10.1186/1475-2875-8-269

**Published:** 2009-11-26

**Authors:** Janet Cox-Singh

**Affiliations:** 1 Division of Cellular and Molecular Medicine, Centre For Infection, St George's University of London, Cranmer Terrace, London SW17 0RE, UK; 2 The Malaria Research Centre, University Malaysia Sarawak, Malaysia

## Abstract

The simian malaria parasite *Plasmodium knowlesi *is transmitted in the forests of Southeast Asia. Symptomatic zoonotic knowlesi malaria in humans is widespread in the region and is associated with a history of spending time in the jungle. However, there are many settings where knowlesi transmission to humans would be expected but is not found. A recent report on the Ra-glai population of southern central Vietnam is taken as an example to help explain why this may be so.

## Background

A recent study on *Plasmodium knowlesi *malaria in Vietnam failed to discover symptomatic *P. knowlesi *in the human population [1]. In their study, the authors used *P. knowlesi *PCR primers to re-examine blood samples collected during two large cross-sectional malaria surveys in the Ninh Thuan province during 2004 and 2005. The population screened was mainly of Ra-glai ethnicity, living in hilly, densely forested areas with frequent overnight stays in the jungle. There are many parallels with the population in Sarawak Malaysian Borneo, where a large number of human cases of zoonotic knowlesi malaria occur annually [2,3]. The authors report that vectors of *P. knowlesi *are in the area, but it is not known if there are macaques or indeed if they carry *P. knowlesi *parasites [4]. Nonetheless, all of the indicators suggest that human cases of *P. knowlesi *could occur in this population, which is, therefore, worthy of screening.

## Discussion

The authors reported that during their surveys 13.6% of 4,000 individuals screened were PCR positive for the human-adapted malaria parasites, PCR for *P. knowlesi *was not included. Of the positives, 210 were *Plasmodium malariae*. Ninety-five of the 210 were selected for re-screening with *P. knowlesi *PCR primers. Three samples, two from children, two and three years of age respectively, and one from a young adult were confirmed *P. knowlesi *PCR-positive. One of the children remained positive with time. The authors conclude that the prevalence of *P. knowlesi *is low and asymptomatic in this population. This finding is not surprising given that the authors re-screened samples PCR-positive for *P. malariae*. *Plasmodium malariae *and *P. knowlesi *are phylogenetically distinct, the one notable similarity is at the morphological level. Indistinguishable morphology between these parasites gave rise to misdiagnosis and masking of symptomatic human cases of *P. knowlesi *malaria in Sarawak, often putting patients lives at risk [3]. Therefore, to screen properly this important population in Vietnam for *P. knowlesi *infection, PCR negative samples found during the primary screen, those that were not amplified by falciparum, vivax, malariae or ovale specific PCR primers, would have been more likely to yield positive results for *P. knowlesi*. Of the three *P. knowlesi *positives reported, one was negative by microscopy and the others were either *P. vivax *or *P. vivax *with *P. falciparum *by microscopy. For this study, the authors used *P. knowlesi *primers Pmk8 and Pmk9 published by the Sarawak research group [3]. These primers have recently been reported to cross-hybridize with a small number of *Plasmodium vivax *isolates in a stochastic (random) manner [5]. Two of the three positive cases had microscopy confirmed *P. vivax*. It is possible that the persistent infection reported was due to cross-hybridization with *P. vivax*, an explanation that would better fit the biology of the two parasites. Taken together with sampling and other problems involving non-specific human DNA amplification reported by Van den Eede *et al*, this account of *P. knowlesi *in Vietnam may not accurately represent the situation there.

## Conclusion

Persistent *P. knowlesi *infections, particularly in very young children would indeed add interest to what is currently known of *P. knowlesi *epidemiology. Cryptic knowlesi infections were reported recently in Thailand [6], but mostly as mixed infections with *P. falciparum *and *P. vivax *and in areas where these human malaria parasites predominate. The cases of *P. falciparum *and *P. vivax *in Sarawak, Malaysian Borneo, are in decline, particularly in areas where symptomatic *P. knowlesi *is reported. *Plasmodium knowlesi *remains zoonotic and by definition less well-adapted to the human host. In areas where *Plasmodium falciparum *and *P. vivax *are prevalent, they would perhaps be expected to out-compete *P. knowlesi *in the human host. It will be interesting to follow the incidence of symptomatic knowlesi malaria in neighbouring countries, particularly when the human-adapted parasites are in decline and where humans continue to utilize forested areas with known knowlesi transmission.

## Competing interests

The authors declares no competing interests.
